# The protective effect of neighbourhood social cohesion on adolescent mental health following stressful life events

**DOI:** 10.1017/S0033291719001235

**Published:** 2020-06

**Authors:** Mila Kingsbury, Zahra Clayborne, Ian Colman, James B. Kirkbride

**Affiliations:** 1 School of Epidemiology and Public Health, University of Ottawa, Ottawa, Canada; 2 Division of Psychiatry, UCL, London, England

**Keywords:** Adolescence, mental health, neighbourhoods, social cohesion, stressful life events

## Abstract

**Background:**

Exposure to stressful life events is an established risk factor for the development of adolescent mental disorder. Growing evidence also suggests that neighbourhood social environments, including strong social cohesion, could have a protective effect on mental health. However, little is known about how neighbourhood social cohesion may buffer against the effects of stressful life events on adolescent mental health. Our aim was to assess whether neighbourhood social cohesion modifies the association between stressful life events and adolescent mental health outcomes.

**Methods:**

Data were drawn from a nationally-representative prospective sample of Canadian adolescents, including 5183 adolescents aged 12/13 years at T1 and 14/15 years at T2. Caregivers reported neighbourhood social cohesion at T1, and exposure to stressful life events between T1 and T2. Symptoms of mental health and behaviour problems were self-reported by adolescents at T1 and T2. Multivariable logistic regression was used to determine whether the relationship between stressful life events and outcomes was modified by neighbourhood social cohesion.

**Results:**

Associations between stressful life events and adolescent outcomes were statistically significantly lower in neighbourhoods with greater social cohesion for: depression/anxiety (high cohesion OR = 0.98 *v.* low cohesion OR = 3.11), suicidal ideation (OR_high_ = 1.30 *v.* OR_low_ = 5.25), aggression/conduct disorder (OR_high_ = 1.09 *v.* OR_low_ = 4.27), and property offence (OR_high_ = 1.21 *v.* OR_low_ = 4.21).

**Conclusions:**

Greater neighbourhood social cohesion appeared to buffer the effects of stressful life events on several domains of adolescent mental health. This potentially presents a target for public health intervention to improve adolescent mental health and behavioural outcomes.

Stressful life events [SLEs] in childhood and adolescence are well-established risk factors for the development of later psychiatric problems (Kessler *et al*., 1997). Exposure to both acute and chronic stressors early in life, ranging from parental separation to exposure to violence or abuse, can have adverse, long-term impacts on mental health. For example, SLEs, including maltreatment, have been linked to increased depressive and anxiety symptoms (Michl *et al*., 2013), as well as antisocial behaviour (Lansford *et al*., 2002), conduct disorder (Jaffee *et al*., 2005), hyperactivity (De Sanctis *et al*., 2012), and suicidal ideation (Afifi *et al*., 2008). These early mental health problems can persist into adulthood (Naicker *et al*., 2013), carrying additional risks of experiencing substance use problems, lower educational attainment, difficulty maintaining stable employment, and difficulty developing healthy and meaningful interpersonal relationships (Colman *et al*., 2009). Given their potential long-term impacts on mental health and psychosocial development, early life SLEs present a major public health issue for which we need to identify potential factors which may improve the long-term outlook for children exposed to these early-life stressors.

Social cohesion, defined as the level of connectedness between individuals living in close geographical proximity (Sampson, 2003), may present such a factor, and has been linked with better physical and mental health (Araya *et al*., 2006; Echeverría *et al*., 2008). One mechanism through which this may operate is via the strength of community ties, which may create environments where health-promoting behaviours are reinforced, and negative behaviours (e.g. vandalism, drinking in public spaces) are discouraged (Kawachi and Berkman, 2001; Echeverría *et al*., 2008). A growing number of studies demonstrate the impact of social cohesion on a number of youth mental health outcomes (De Silva *et al*., 2005; Donnelly *et al*., 2016). In particular, inverse relationships have been demonstrated between social cohesion and child and adolescent depression (De Silva *et al*., 2005; Echeverría *et al*., 2008; Kingsbury *et al*., 2015; Donnelly *et al*., 2016; Solmi *et al*., 2017), anxiety (De Silva *et al*., 2005; Kingsbury *et al*., 2015; Donnelly *et al*., 2016), and externalizing behaviours (Curtis *et al*., 2004; De Silva *et al*., 2005; Jaffee *et al*., 2007).

Emerging research has also demonstrated that residing in highly cohesive neighbourhoods may strengthen a child's ability to positively cope with adversity (Silk *et al*., 2004; Jaffee *et al*., 2007). For example, among children exposed to maltreatment, those who report stronger social ties with adults in their community, including their parents, extended family members, and schoolteachers, tend to report better overall adjustment compared with children who report weaker social ties with community members (Cicchetti and Rogosch, 1997; Jaffee *et al*., 2007). Children with stronger community ties also score more highly on measures of resiliency, and do not present with the elevated levels of antisocial behaviour typically seen among children exposed to maltreatment (Jaffee *et al*., 2007). This raises the possibility that for children and adolescents exposed to SLEs, living in a cohesive community may buffer the adverse, long-term impacts of these stressors. To our knowledge, however, no population-based, longitudinal study has tested whether social cohesion moderates the relationship between childhood adversity and subsequent common mental and behavioural disorders. We therefore sought to investigate the role of neighbourhood social cohesion as a potential modifier of the associations between exposure to stressful life events in early adolescence and symptoms of mental and behavioural disorders two years later, using well-characterized longitudinal data from a large, prospective cohort. We hypothesized that neighbourhood social cohesion would buffer the effect of stressful life events on negative outcomes in adolescence.

## Methods

### Data source

Data for the present study were drawn from cycles 5 (2001–2002), 6 (2003–2004), 7 (2005–2006), and 8 (2007–2008) of the National Longitudinal Survey of Children and Youth (NLSCY). The NLSCY is a longitudinal study of Canadian children and adolescents designed to track multiple aspects of youth health and development. Stratified sampling resulted in a sample that is considered representative of children living in private homes in Canada's 10 provinces (excluding families in the Armed Forces, those living in institutions, on reserves, and in the northern Territories). Cohort members were followed prospectively, with assessments from multiple informants every two years. Statistics Canada obtained written informed consent from parents of survey respondents and regulates access of survey data through National research data centres. The present sample was based on 5913 respondents who were aged 12/13 (T1) in cycles 5, 6, or 7, and for whom data were available 2 years later at ages 14/15 (T2), during cycles 6, 7, or 8.

### Measures

#### Mental and behavioural disorders

Adolescent psychiatric symptoms were self-reported at T1 and T2 using behaviour scales adapted from questionnaires used in the Montreal Longitudinal Study and the Ontario Child Health Study (Boyle *et al*., 1987). The scales were designed to identify children who would be most likely to meet DSM-III-TR criteria for a psychiatric diagnosis. Paper questionnaires were completed privately by adolescents, and returned to interviewers in a sealed envelope. For the present study, the following subscales were of interest: *anxiety/depression* (7 items, e.g. ‘I am not as happy as other people my age’; ‘ I am too fearful or nervous’), *physical aggression*/*conduct disorder* (6 items, e.g. ‘I get into many fights’), *property offence* (6 items, e.g. ‘I vandalize’), and *hyperactivity/inattention* (7 items, e.g. ‘I am easily distracted’; ‘I am impulsive, I act without thinking’). Adolescents responded on a 3-point scale (‘never or not true’; ‘sometimes or somewhat true’; ‘often or very true’). Subscale scores, pro-rated for item-level missingness, were provided by Statistics Canada. For each outcome, subscale scores were dichotomized at the top decile to indicate psychopathology of potential clinical relevance, consistent with previous studies (McMartin *et al*., 2014; Kingsbury *et al*., 2015).

Adolescent suicidal behaviour was assessed at T2 on the basis of two questions. First, adolescents were asked whether they had seriously considered suicide in the past 12 months, with *suicidal ideation* defined as answering ‘yes’ to this question. Second, adolescents who screened positive for *suicidal ideation* were additionally asked how many times they had attempted suicide in the past year. For the present study, *suicide attempt* was defined as one or more attempts in the past year.

#### Stressful life events [SLEs]

Adolescent exposure to SLEs in the past 2 years was reported by the person most knowledgeable about the child at T2 (hereafter referred to as ‘primary caregiver’; approximately 90% were biological mothers). Respondents were asked whether the participant had experienced an event that caused the participant ‘a great amount of worry or unhappiness’ in the past 2 years (i.e. since baseline). Those who answered ‘yes’ were then asked about 13 specific life events (e.g. parental death, parental divorce/separation, abuse or fear of abuse). For the present study, we defined exposure to SLEs in the previous two years as a binary variable (any *v.* none).

#### Neighbourhood social cohesion

Primary caregivers reported on neighbourhood social cohesion at T1. The social cohesion score was based on 5 statements, rated on a 4-point scale from ‘strongly agree’ to ‘strongly disagree’ (‘people around here are willing to help their neighbours’; ‘there are adults in the neighbourhood that children can look up to’; ‘when I'm away from home, I know that my neighbours will keep their eyes open for possible trouble’; ‘you can count on adults in this neighbourhood to watch out that children are safe and don't get in trouble’; ‘if there's a problem around here, the neighbours get together to deal with it’). Scores for these items were summed to create a total score for social cohesion, ranging from 0–15. For the present study, social cohesion was dichotomized at the first quartile (i.e. comparing those in low-cohesion neighbourhoods to all others).

#### Covariates

Adolescent *sex* was reported by the primary caregiver at T1.

Caregivers reported on adolescent *ethnicity* (white/non-white) at T1. When possible, data from earlier cycles was carried forward by Statistics Canada to replace missing data on this variable.

Depressive symptoms in the primary caregiver were assessed at T1 using the Depression Rating Scale, a shortened 12-item version of the CES-D (Radloff, 1977). For the present study, *caregiver depression* was operationalized as a score in the top 10% on the depression scale.

Family *poverty* was assessed using the ratio of income to the corresponding low-income cut-off (LICO). LICO is defined as the income below which a family would have difficulty making ends meet, and is based on family size and geographic area (Statistics Canada, 2009). For the present analyses, this ratio was dichotomized at 1 (i.e. comparing families with incomes below and above the low-income cut-off).

Family composition was reported by the primary caregiver at T1. For the present analysis, we dichotomized this variable to consider adolescents living with two *biological parents v.* those living in other family structures (e.g. step-parent families, single-parent families, foster families).

Primary caregivers reported on their levels of *social support* using the 8-item social support scale. Caregivers rated their agreement with each item (e.g. ‘There are people I can count on in an emergency’) on a 4-point scale, from ‘strongly agree’ to ‘strongly disagree’. For these analyses, social support was dichotomized at the bottom quartile.

Finally, perceived *neighbourhood safety* was assessed in the NLSCY using a 3-item scale. Caregivers rated their agreement with each statement (‘It is safe to walk alone in this neighbourhood after dark’; ‘It is safe for children to play outside during the day’; ‘There are safe parks, playgrounds, and play spaces in this neighbourhood’) on a 4-point scale, from ‘strongly agree’ to ‘strongly disagree’. Scores on neighbourhood safety were trichotomized to reflect low (bottom 25%), average (middle 50%), and high (top 25%) safety.

### Analysis

Separate multivariable logistic regression models were estimated for each mental health outcome. First, we established whether SLE exposure was associated with each outcome by fitting a model including baseline mental illness symptoms, stressful life events, neighbourhood social cohesion, ethnicity, sex, caregiver depression and family poverty. *p* < 0.05 was considered to be statistically significant. To test the modifying effect of neighbourhood social cohesion on the association between SLE exposure and each outcome, we fitted an interaction term between SLE exposure and social cohesion, and tested whether this improved model fit via Score χ^2^ tests. In the presence of effect modification, we reported stratified effects of SLEs on each outcome, in low and higher social cohesion neighbourhoods, as defined above. Normalized survey weights based on derived weights generated by Statistics Canada were used to take into account the complex survey design. Cases with missing data on the exposure (SLEs) or effect modifier (neighbourhood cohesion) were listwise deleted. All analyses were conducted using SAS software (version 9.3, SAS Institute Inc., Cary, NC, USA).

## Results

Adolescents who had experienced SLEs in the past 2 years were more likely to be white, have a depressed primary caregiver, have a family income below the corresponding low-income cut-off, and were less likely to be living with two biological parents (Table 1).

**Table 1.** Sample characteristics by exposure to stressful life events (SLEs) (weighted percentages)^a^

	Total sample	No SLEs in past 2 years	1 or more SLE in past 2 years	χ^2^	*p* value
	*N* = 5913	*n* = 4567 (78.7%)	*n* = 1320 (21.3%)		
Sex (female)	48.7%	48.7%	48.7%	0.002	0.968
Child ethnicity (non-white)	9.40%	10.63%	4.89%	36.51	<0.001
Primary caregiver depressed	9.05%	7.90%	13.31%	34.22	<0.001
Below low income cut-off	12.83%	11.79%	16.67%	21.09	<0.001
Living with two biological parents	69.21%	71.21%	61.85%	40.86	<0.001
Low social support	12.61%	12.52%	12.96%	0.17	0.678
Low neighbourhood safety	17.25%	17.08%	17.88%	1.81	0.405
Low neighbourhood cohesion	16.07%	16.33%	15.05%	1.02	0.312

a Raw frequencies in each cell are not given, in accordance with Statistics Canada guidelines

### Predicting adolescent mental health

**Fig. 1.**
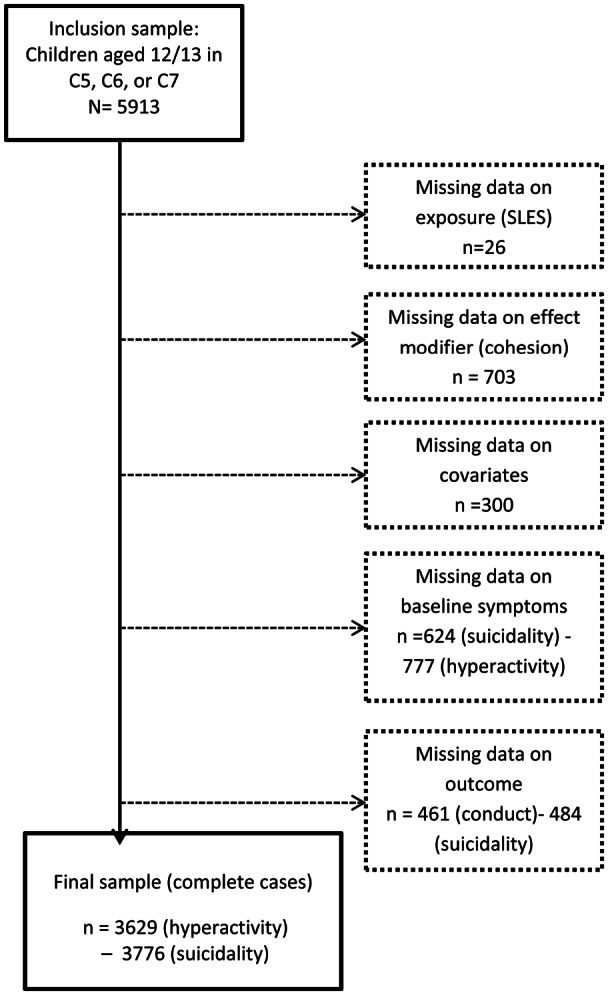
Sample size flow diagram.

The available sample size with complete data varied depending on the outcome investigated, from 3629 for hyperactivity to 3776 for suicidality (Fig. 1). Those missing data on T1 variables (cohesion, baseline mental health symptoms) were more likely to be male, non-white, living with a depressed caregiver, living in poverty, in low safety neighbourhoods, have a caregiver with low social support, and less likely to be living with 2 biological parents (online Supplementary eTable S1). Those who dropped out between T1 and T2 were more likely to be male, white, and to live in neighbourhoods characterized by low cohesion and low safety (online Supplementary eTable S2).

#### Depression/anxiety

In our main model, living in a cohesive neighbourhood was protective against adolescent depression/anxiety (OR 0.62, 95% CI 0.43–0.90; Table 2). There was a significant interaction between SLEs and neighbourhood cohesion (χ^2^ = 14.98, *p* < 0.001; Table 3). In low-cohesion neighbourhoods, SLEs were significantly and positively associated with adolescent depression/anxiety (OR 3.11, 95% CI 1.64–5.90), but no effect was observed in higher cohesion neighbourhoods (OR 0.99, 95% CI 0.71–1.37).

**Table 2.** Results of logistic regression predicting adolescent mental health outcomes

	Depression/anxiety			Suicidal ideation			Suicide attempt			Conduct disorder			Property offence			Hyperactivity		
	OR	95% CI		OR	95% CI		OR	95% CI		OR	95% CI		OR	95% CI		OR	95% CI	
		low	high		low	high		low	high		low	high		low	high		low	high
Stressful life events	1.26	0.95	1.67	**1**.**54**	**1**.**14**	**2.07**	**1**.**74**	**1**.**16**	**2**.**59**	**1**.**44**	**1.08**	**1.91**	**1**.**50**	**1**.**14**	**1**.**86**	1.10	0.85	1.43
Neighbourhood cohesion	**0.62**	**0.43**	**0.90**	0.82	0.55	1.21	1.30	0.80	2.11	1.20	0.85	1.70	0.99	0.72	1.36	0.93	0.68	1.26
Female sex	**2.81**	**2.15**	**3.66**	**2.35**	**1.77**	**3.12**	**3.53**	**2.29**	**5.45**	**0.58**	**0.43**	**0.72**	**0.73**	**0.59**	**0.91**	1.09	0.88	1.36
Child ethnicity (non-white)	**1.63**	**1.12**	**2.37**	0.76	0.45	1.29	0.91	0.47	1.79	1.39	0.93	2.08	0.75	0.49	1.12	**2.15**	**1.54**	**3.01**
Caregiver depression	**1.69**	**1.15**	**2.48**	1.03	0.65	1.64	1.11	0.60	2.07	1.40	0.93	2.09	1.32	0.92	1.89	**2.43**	**1.75**	**3.37**
Poverty	0.71	0.47	1.08	0.64	0.40	1.02	**0.30**	**0.14**	**0.66**	**1.60**	**1.211**	**2.32**	**1.42**	**1.02**	**1.97**	1.31	0.94	1.82
Family structure (two biological parents)	0.86	0.65	1.12	**0.52**	**0.39**	**0.69**	**0.42**	**0.29**	**0.63**	0.85	0.64	1.13	**0.69**	**0.55**	**0.88**	**0.76**	**0.59**	**0.97**
Low social support	1.00	0.70	1.44	0.70	0.45	1.09	**0.48**	**0.25**	**0.95**	0.66	0.43	1.01	0.93	0.66	1.30	1.01	0.73	1.39
Average neighbourhood safety (ref: low)	1.05	0.75	1.47	1.16	0.78	1.71	0.99	0.60	1.65	1.39	0.96	2.02	**1.63**	**1.16**	**2.28**	**6.79**	**5.24**	**8.80**
High neighbourhood safety (ref: low)	0.66	0.43	1.02	0.84	0.52	1.36	0.65	0.33	1.25	1.01	0.64	1.60	1.39	0.94	2.07	1.11	0.75	1.64
High time 1 symptoms	**5.48**	**4.07**	**7.38**	**11.11**	**7.94**	**15.55**	**9.98**	**5.83**	**17.10**	**7.96**	**5.99**	**10.57**	**7.01**	**5.37**	**9.17**	**6.79**	**5.24**	**8.80**

OR, Odds ratio; 95% CI, 95% confidence intervals.

*Note*: Statistically significant effects are shown in bold.

**Table 3.** Effects of stressful life events on adolescent mental health outcomes, stratified by level of neighbourhood cohesion^a^

			Low neighbourhood cohesion			Higher neighbourhood cohesion		
	SLE*cohesion interaction			(95% CI)			(95% CI)	
	Score χ^2^	*p* value	OR	lower	upper	OR	lower	upper
Depression/Anxiety	**14.98**	<0.001	**3**.**11**	**1.64**	**5.90**	0.99	0.71	1.37
Suicidal ideation	**7.52**	**0.006**	**5.25**	**2.28**	**12.08**	1.30	0.94	1.81
Suicide attempt	2.79	0.094	**3.02**	**1.24**	**7.37**	1.44	0.90	2.29
Conduct disorder	**10.90**	**0.001**	**4.27**	**2.23**	**8.19**	1.09	0.78	1.52
Property offence	**8.68**	**0.003**	**4.21**	**2.28**	**7.76**	1.21	0.92	1.60
Hyperactivity	0.27	0.60	1.01	0.53	1.93	1.15	0.87	1.54

95% CI, 95% confidence interval.

*Note*: significant χ^2^s and odds ratios (ORs) are presented in bold.

a Adjusted for child sex, ethnicity, caregiver depression, and family poverty.

#### Suicidal ideation

Adolescents who had experienced SLEs in the past two years were more likely to report suicidal ideation than those who had not (OR 1.54, 95% CI 1.14–2.07). However, this main effect was superseded by a significant interaction between SLEs and neighbourhood cohesion (χ^2^ = 7.52, *p* = .006); thus, the effect of SLEs on adolescent suicidal ideation was substantially greater in low social cohesion neighbourhoods (OR 5.25, 95% CI 2.23–1.32) than higher cohesion neighbourhoods (OR 1.30, 95% CI 0.94–1.81, Table 3).

#### Suicide attempt

Similarly to our findings for suicidal ideation, adolescents who had experienced SLEs in the past two years were also more likely to have attempted suicide than those who had not (OR 1.74, 95% CI 1.16–2.59). The interaction between SLEs and neighbourhood social cohesion was not statistically significant (χ^2^ = 2.79, *p* = 0.094; Table 3); however, inspection of the stratified results suggested that SLEs had a significant effect on suicide attempt among adolescents in low cohesion neighbourhoods (OR 3.02, 95% CI 1.24–7.37) but not higher cohesion neighbourhoods (OR 1.44, 95% CI 0.90–2.29).

#### Aggression/conduct disorder

Adolescents who had experienced SLEs had higher odds of elevated aggressive/conduct symptoms (OR 1.44, 95% CI 1.08–1.91). The interaction between SLEs and neighbourhood cohesion was also statistically significant (χ^2^ = 10.90, *p* < 0.001), and as for most other outcomes, the effect of SLEs was stronger in low-cohesion neighbourhoods (OR 4.49, 95% CI 2.23–8.19), but absent in higher cohesion neighbourhoods (Table 3).

#### Property offence

Similarly, the experience of SLEs was associated with increased risk of property offence (OR 1.50, 95% CI 1.14–1.86), with strong evidence of an interaction between SLEs and neighbourhood cohesion (χ^2^ = 8.68, *p* = .003) suggesting that this association was significantly stronger in low social cohesion neighbourhoods (OR 4.21, 95% CI 2.28–7.76) than in higher social cohesion neighbourhoods (OR 1.21, 95% CI 0.92–1.60, Table 3).

#### Hyperactivity

Neither stressful life events, neighbourhood social cohesion, nor their interaction significantly predicted adolescent hyperactivity (Tables 2 and 3).

### Sensitivity analysis

We conducted a *post hoc* sensitivity analysis exploring the effects of exposure to SLEs across three levels of neighbourhood social cohesion (bottom 25%; middle 50%; top 25%). Results (online Supplementary eTable S3) suggested that the moderating effects of social cohesion were largely constrained to the lowest quartile: SLEs were significantly associated with mental health and behavioural outcomes at the lowest levels of social cohesion, but not in moderate or high cohesion neighbourhoods.

## Discussion

The association between SLEs and four out of six major mental health or behavioural outcomes in young adolescents was stronger amongst those living in low social cohesion neighbourhoods than higher social cohesion neighbourhoods measured two years earlier in this longitudinal cohort study. A trend to this effect was found for a fifth outcome, suicide attempts, although no discernable effects were apparent for our final outcome, hyperactivity. Associations between exposure to SLEs and psychiatric symptoms were attenuated to the null for adolescents living in neighbourhoods with higher levels of social cohesion. These results could not be explained by differences in income, sex, ethnicity, family structure, social support, neighbourhood safety, mental health at baseline, or depression in the primary caregiver. The consistency of our results suggest that neighbourhood social cohesion may effectively buffer children and adolescents from the otherwise potentially deleterious effects that SLEs can have on future mental health and behavioural problems. If causal, our findings strongly suggest that efforts to improve neighbourhood social cohesion, specifically here, amongst teenagers, will have positive effects on future mental health.

### Neighbourhood social cohesion and mental health

Social cohesion was, on its own, associated with only one of the adolescent mental health outcomes assessed – symptoms of depression and anxiety. Across the majority of outcomes, higher social cohesion appeared to buffer the effects of exposure to SLEs. Among adolescents residing in neighbourhoods characterized by low social cohesion, the recent experience of SLEs was associated with increased risk of depression/anxiety, suicide ideation and attempt, aggression/conduct disorder, and property offence. In higher cohesion neighbourhoods, the effects of SLEs on these psychiatric symptoms were attenuated to the null. Exposure to SLEs in childhood and adolescence has been consistently linked to later psychiatric illness (Kessler *et al*., 1997; Anda *et al*., 2006; Schilling *et al*., 2007), including internalizing(Chapman *et al*., 2004; Schilling *et al*., 2007) and externalizing problems (Schilling *et al*., 2007; Baglivio *et al*., 2014), and suicidal behaviour (Isohookana *et al*., 2013). Few studies have previously investigated whether neighbourhood social cohesion buffers such stressors, although one study showed that higher perceived neighbourhood cohesion attenuated the effects of maternal hostility on child externalizing behaviours, including symptoms of conduct disorder and property offences (Silk *et al*., 2004). Two further studies from the same sample have shown that greater neighbourhood social cohesion moderates the effects of childhood poly-victimization on early and late adolescent psychotic symptoms (Crush *et al*., 2018*a*, 2018*b*). Our results suggest similar mechanisms may be at play with regards to neighbourhood social cohesion.

At the individual level, social support has long been hypothesized to buffer against the effects of stress (Cohen and Wills, 1985) on mental health. Evidence suggests this may operate in at least two ways (Kawachi and Berkman, 2001). First, the perceived availability of social support can lead to more benign cognitive appraisals of stressors as they are encountered, and second, the experience of social support during a time of stress can lead to a dampening of the behavioural and even physiological responses to stressors (Kawachi and Berkman, 2001). Similar mechanisms may apply to adolescents living in socially cohesive neighbourhoods following exposure to an SLE. Notably, our results were not explained by caregiver social support at the individual level, suggesting that social processes operating at the wider neighbourhood environment may be at play. It is also possible that those living in more socially cohesive neighbourhoods benefit from social learning via increased exposure to multiple adult role models, or more generally from positive emotional and instrumental support between neighbours (Silk *et al*., 2004). Our findings suggest further longitudinal research is warranted to tease out potential pathways between SLEs, social cohesion and adolescent mental health.

### Alternate interpretations

Results of sensitivity analysis suggested that the moderating effects of social cohesion were most pronounced for children living in neighborhoods with the lowest levels of social cohesion; that is, there may be a threshold of social cohesion above which additional incremental improvements have little effect on resilience. Alternately, these results can be viewed as evidence for a ‘double disadvantage’ effect, whereby the deleterious effects of life stressors on mental health are only evident among adolescents additionally exposed to suboptimal neighbourhood conditions. Beyond social cohesion, other neighbourhood factors have also been reported to moderate the associations between acute SLEs and psychiatric outcomes, but only below certain thresholds; for example, strong associations between SLEs and increased aggression appear to be restricted to children living in the most economically disadvantaged neighbourhoods (Attar *et al*., 1994). Importantly, our findings were impervious to adjustment for neighbourhood safety, lending credence to the possibility that neighbourhood social cohesion had moderating effects on various mental health outcomes following exposure to SLEs, over and above the influence of neighbourhood structural disadvantage. Whether these results are interpreted as a buffering effect of higher levels of social cohesion, or an amplification of the negative effects of SLEs by low social cohesion, they nonetheless suggest that improving low social cohesion may have beneficial consequences for youth exposed to life stress.

### Strengths and limitations

We acknowledge some limitations to the present study. Caregiver report of neighbourhood social cohesion may reflect certain aspects of their personality and behaviour, introducing bias. For example, parents who are actively involved in the community may also be more likely to promote adaptive behaviour in their children. We sought to minimise this by adjusting for caregiver depression and individual-level social support, however, we were unable to control for other aspects of the primary caregiver's mental health and behaviour, including parenting practices. Adolescent psychiatric symptoms were assessed using self-report scales, and as such may not reflect psychiatric diagnoses. Although these scales were designed to correspond to DSM-III criteria, they are not intended as diagnostic instruments. SLEs were assessed via retrospective caregiver report between T1 and T2. While such data are potentially subject to recall bias (Colman *et al*., 2016), it has been suggested that self reports can be reliable for relatively rare and important events (i.e. death, divorce) (Schwarz, 2007). The short timeframe for recall in the present study also increases confidence in the reliability of the reports. For certain events (i.e. abuse, fear of abuse or parental alcohol abuse), caregivers may have withheld information out of fear of recrimination or social desirability. Future studies may need to use a multi-informant approach to assess exposure to stressful life events in a more objective way. Finally, we did not differentiate between the 13 different types of stressors assessed. However, studies examining multiple types of adverse childhood experiences have reported largely non-specific effects on mental health (Schilling *et al*., 2007).

These limitations were balanced by notable strengths. Our study leveraged data from a large population-based prospective sample of adolescents. Additionally, the use of prospectively collected data and adjustment for baseline symptoms allowed for clarity in the temporality of relationships between neighbourhood cohesion and mental health.

### Public health implications

The consistency of our findings strengthens the possibility that neighbourhood cohesion in early adolescence may mitigate mental health problems for teenagers exposed to stressful life events in childhood. Given adolescence is a key period for the emergence of mental health disorders (Patton *et al*., 2016), which often predicts worse adulthood physical, mental and social outcomes (Naicker *et al*., 2013; Patton *et al*., 2014), identifying modifiable prevention targets is a central public mental health concern. We suggest that selected intervention strategies to promote social integration amongst youth who have recently experienced SLEs could be warranted, particularly given that over 1 in 5 adolescents in our sample had experienced at least one SLE in the two years before assessment. These could include helping such individuals develop and maintain peer relationships, known to be of central importance to adolescent health and well-being (Patton *et al*., 2016), or by establishing or enhancing school- or community-based initiatives which promote conditions for greater prosocial behaviours (van den Bos *et al*., 2018) and social cohesion (Donnelly *et al*., 2016).

## Supplementary material

For supplementary material accompanying this paper visit https://doi.org/10.1017/S0033291719001235. click here to view supplementary material
