# Supplementary material for: The protective effect of neighbourhood social cohesion on adolescent mental health following stressful life events
Source: Psychol Med. 2019 Jun 10;50(8):1292–9. doi: 10.1017/S0033291719001235 (PMC7322549; doi:10.1017/S0033291719001235)
Supplement: Supplementary file 1 [file S0033291719001235sup001.docx]

**eTable 1. Missing data analysis comparing those missing T1 data to those with complete data (weighted percentages)^a^.**

|  | **Missing T1 data** | **Has T1 data** | ***Χ*^2^** | **p-value** |
| --- | --- | --- | --- | --- |
| **Male** | 54.24 % | 50.02 % | 8.88 | 0.003 |
| **Child ethnicity (non-white)** | 12.26 % | 8.17 % | 23.17 | < 0.0001 |
| **Primary caregiver depressed** | 11.49 % | 8.00 % | 17.74 | < 0.0001 |
| **Below low income cut-off** | 14.77 % | 12.03 % | 8.35 | 0.004 |
| **Living with biological parents** | 61.58 % | 72.38 % | 68.07 | < 0.0001 |
| **Low neighbourhood safety** | 19.28 % | 16.49 % | 25.90 | < 0.0001 |
| **Low caregiver social support** | 14.84 % | 11.61 % | 11.82 | < 0.001 |

^a^ Raw frequencies in each cell are not given, in accordance with Statistics Canada guidelines

**eTable 2. Dropout analysis comparing those with complete data to those who dropped out between T1 and T2 (weighted percentages)^a^.**

|  | **Dropout from T1 🡪 T2** | **In final sample** | ***Χ*^2^** | **p-value** |
| --- | --- | --- | --- | --- |
| **Male** | 54.22 % | 49.34 % | 4.68 | 0.030 |
| **Child ethnicity (non-white)** | 6.87 % | 8.38 % | 1.43 | 0.023 |
| **Primary caregiver depressed** | 9.31 % | 7.78 % | 1.54 | 0.215 |
| **Below low income cut-off** | 14.21 % | 11.68 % | 2.98 | 0.084 |
| **Living with biological parents** | 74.24 % | 72.09 % | 1.138 | 0.286 |
| **Low caregiver social support** | 9.64% | 11.93% | 2.508 | 0.113 |
| **Low neighbourhood safety** | 22.55% | 15.54% | 17.51 | < 0.001 |
| **Low neighbourhood cohesion** | 20.40 % | 15.48 % | 8.80 | 0.003 |

^a^ Raw frequencies in each cell are not given, in accordance with Statistics Canada guidelines

**eTable3.** **Sensitivity analysis testing the effects of exposure to SLEs across three levels of neighbourhood social cohesion.**

|  | **SLE*cohesion interaction** | | **Low Neighbourhood Cohesion** | | | **Moderate Cohesion** | | | **High Cohesion** | | |
| --- | --- | --- | --- | --- | --- | --- | --- | --- | --- | --- | --- |
|  | Score *Χ*^2^ | p-value | OR | (95% CI) | | OR | (95% CI) | | OR | (95% CI) | |
|  |  |  |  | lower | upper |  | lower | upper |  | lower | upper |
| Depression/Anxiety | **16.08** | **< 0.001** | **3.11** | **1.64** | **5.89** | 1.21 | 0.78 | 1.61 | 0.76 | 0.35 | 1.68 |
| Suicidal Ideation | **7.91** | **0.019** | **5.25** | **2.28** | **12.08** | 1.30 | 0.88 | 1.92 | 1.41 | 0.74 | 2.71 |
| Suicide Attempt | 2.71 | 0.259 | **3.02** | **1.24** | **7.37** | 1.48 | 0.87 | 2.53 | 1.54 | 0.56 | 4.25 |
| Conduct Disorder | **11.18** | **0.004** | **4.27** | **2.23** | **8.19** | 1.15 | 0.78 | 1.68 | 0.78 | 0.38 | 1.61 |
| Property Offence | **8.69** | **0.013** | **4.21** | **2.28** | **7.76** | 1.21 | 0.88 | 1.67 | 1.19 | 0.69 | 2.04 |
| Hyperactivity | 0.61 | 0.738 | 1.01 | 0.53 | 1.93 | 1.13 | 0.81 | 1.58 | 1.33 | 0.73 | 2.40 |
